# A dataset of alternately located segments in protein crystal structures

**DOI:** 10.1038/s41597-024-03595-4

**Published:** 2024-07-17

**Authors:** Aviv A. Rosenberg, Ailie Marx, Alexander M. Bronstein

**Affiliations:** 1 Department of Computer Science, Technion – Israel Institute of Technology, Haifa, Israel; 2 Department of Molecular and Computational Biosciences and Biotechnology, Migal – Galilee Research Institute, Qiryat, Israel

**Keywords:** X-ray crystallography, Data mining

## Abstract

Protein Data Bank (PDB) files list the relative spatial location of atoms in a protein structure as the final output of the process of fitting and refining to experimentally determined electron density measurements. Where experimental evidence exists for multiple conformations, atoms are modelled in alternate locations. Programs reading PDB files commonly ignore these alternate conformations by default leaving users oblivious to the presence of alternate conformations in the structures they analyze. This has led to underappreciation of their prevalence, under characterisation of their features and limited the accessibility to this high-resolution data representing structural ensembles. We have trawled PDB files to extract structural features of residues with alternately located atoms. The output includes the distance between alternate conformations and identifies the location of these segments within the protein chain and in proximity of all other atoms within a defined radius. This dataset should be of use in efforts to predict multiple structures from a single sequence and support studies investigating protein flexibility and the association with protein function.

## Background & Summary

The diffraction pattern produced from the interaction of X-rays with protein molecules can be used to calculate an electron density map into which a model of the structure can be built and refined. Since the signal from a single molecule is far too weak for detection, proteins are first coerced, through crystallization, into an ordered, repeating lattice of molecules so that the diffraction from each molecule will constructively intensify the signal. Flexible and variable regions of the protein chain will lead to destructive interference, the absence of definable electron density and regions of the protein structure that cannot be modelled, at the extreme, or smeared electron density and a low model certainty, as represented by a high temperature factor otherwise known as the B-factor^1^. Rigid regions of the protein, having little variability between molecules in the crystal lattice, are characterised by high model certainty and low B-factor. The molecules in the crystal can also adopt several well-defined conformations such that the electron density is best described by modelling atoms into two or more discrete locations, commonly termed alternate locations (altlocs).

Proteins are dynamic molecules, transitioning between ensembles of conformations (recently reviewed in^2^), and structural biology has recently been calling for methods that will push beyond the one sequence, one structure framework towards description and prediction of ensembles of conformations^3^. Although the conformationally explored space in crystals is limited by the constraints of the crystal packing, a largely overlooked resource for experimentally observed protein flexibility are X-ray crystal structures modelled with alternate conformations. Gutermuth *et al*. recently argued this resource may have remained unnoticed since to date most modelling approaches either ignore altlocs altogether or resolve them with simple heuristics^4^. Indeed, whilst common structural visualization programs such Chimerax or Pymol display alternate side chain locations, only a single, default backbone conformation is shown unless the user specifically calls up the hidden alternate conformation. In some cases, even tools dedicated to question of protein flexibility appear to overlook alternate conformations. The PDBFlex database was developed to provide information of protein flexibility as revealed through comparison of different PDB structures for the same protein^5^. Looking into the database, last updated in 2020, we were unable to find reference to or use of alternate conformations in single crystal protein structures. More recently a group used machine learning to predict same sequence structure ensembles, again guided by cases of different PDB structures displaying conformational differences and originating from the same sequence, overlooking single crystal protein structures^6^. Altogether it seems that single crystal alternate backbone conformations are underused in attempts to characterize protein ensembles perhaps because the abundance of stable alternate conformations remains underrecognized.

The last years have seen efforts to develop tools for uncovering unmodeled alternately located atoms^7–10^, particularly relevant for older PDB entries before the development of refinement software capable of automatically detecting altlocs. However, there are few resources that collate data on altlocs, from across the PDB. One that does is the Alternate Location Server, part of the OCA browser-database for protein structure/function, that maintains an up-to-date list of PDB structures having alternate locations^11^. Although this list highlights the abundance of altlocs, including in backbone atoms, it provides very minimal data: a single line entry for each structure, noting the total number of residues modelled as altocs and the minimum, maximum and average distance between any backbone altlocs. To provide a more detailed resource useful for surveying the alternate conformation landscape and analyzing their prevalence in greater detail, we have created a custom dataset of alternately modelled backbone segments. The dataset is available through Harvard Dataverse^12^.

## Methods

### Raw data collection

Protein structures are collected from the Protein Data Bank (PDB) through a structured query against the polymer entity data API^13,14^. We queried for all entities in structures meeting the following criteria: (i) Method: X-Ray Diffraction; (ii) X-Ray Resolution ≤ 3.5 Å; (iii) R_free_ ≤ 0.33, (iv) Number of chains ≤ 20.

Each query result contains a list of PDB IDs with an entity number (e.g. 1ABC:1) matching the query criteria, and each entity within a PDB structure corresponds to one or more identical polypeptide chains which exist in the structure. Note that a structure may have more than one unique entity (e.g., 1ABC:1 and 1ABC:2) in which case we would obtain both. For each unique entity ID, we then obtain its associated chains (e.g., 1ABC:A and 1ABC:C), and include all of them in the dataset. In cases where the chain ID in the PDB files (author chains) do not match the canonical chain IDs assigned by the PDB, we map between the author and PDB chains, such that our dataset will contain only the canonical IDs.

### Altloc collection

We use BioPython^15^ to parse the PDB structure files and extract the residues and atom locations from the collected chains. For each atom in the structure, we parse all available alternate locations (altlocs) from the file. The altlocs are usually labelled with capital letters starting from ‘A’. In cases where a structure has a non-standard altloc labelling, we sort the labels lexicographically and relabel them starting from ‘A’. In such cases, the dataset will denote these altlocs as e.g. ‘A(Z)’ in the altloc name columns, meaning that the altloc with original label Z is denoted by A in the dataset’s other columns. This relabeling helps keep the column names consistent across different structures.

### Aligning to uniprot sequences

We align the amino acid sequence of each chain to the Uniprot^16^ record sequence to provide a Uniprot index for each residue in the chain. We query the PDB’s entry data API^14^ and examine the metadata to construct a mapping from the specific chain to a list of Uniprot IDs. Whilst most chains map to a single Uniprot ID, there are cases of synthetic proteins which have no associated Uniprot ID, and other cases where a chain is *chimeric* i.e. contains sections from multiple different proteins. We discard such cases and keep only chains which map to a unique Uniprot ID. The alignment is performed using BioPython’s default pairwise alignment algorithm. We used BLOSUM80 as the substitution matrix for the alignment, a gap-opening penalty of $-$10 and a gap-extension penalty of −0.5.

### Backbone locations and dihedral angles per altloc

For each PDB chain, we calculate the backbone angles $\left(\varphi ,\psi \right)$, per altloc. To calculate a dihedral angle at altloc *X*, we take the *X*-altloc coordinate of all atoms participating in the calculation (from the current and previous/next residue). In case the atoms required for dihedral angle calculation from either the previous or next residue do not have the current altloc, we use the single set of coordinates modelled at that location in the calculation of the dihedral angles of all the current altlocs. The dataset also always includes the dihedral angles calculated with all atoms at their default positions, i.e. ignoring altlocs. For each of the backbone atoms in each residue, we also collect its XYZ coordinates under each of the altlocs which exist for it. Finally, we use DSSP to assign a secondary structure per residue.

### B-factors, location standard deviations and distances between altlocs

We calculate the b-factor per residue, by averaging the b-factors of the N, CA and C backbone atoms. This is performed using the default atom positions, and additionally for each altloc which is defined for all three atoms.

For the CA atom, we also calculate, per altloc, the standard deviation in its location and distance from other altlocs, in Angstroms. The standard deviation is obtained from the b-factor ${B}_{X}$ of altloc *X* by ${\sigma }_{X}=\sqrt{{B}_{X}/8{\pi }^{2}}$. For each pair of altlocs *X* and *Y*, we then calculate the distance in Angstroms between the alpha-carbons, ${d}_{X,Y}=\Vert {{\boldsymbol{p}}}_{CA,X}-{{\boldsymbol{p}}}_{CA,Y}\Vert$, where ${{\boldsymbol{p}}}_{CA,X}$ is the location of the alpha-carbon under altloc *X*. We also calculate this distance in units of the standard deviation, which is given by ${\tilde{d}}_{X,Y}=\Vert {{\boldsymbol{p}}}_{CA,X}-{{\boldsymbol{p}}}_{CA,Y}\Vert /\sqrt{{\sigma }_{X}{\sigma }_{Y}}$.

Finally, we calculate the peptide bond length between adjacent residues under each pair of altlocs of the current residue’s carbon and the next residue’s nitrogen.

### Contacts

We calculate the contacts between all atoms of a residue, under all its altlocs, and all other atoms in the PDB structure, also under all possible altlocs. The per-atom contacts are then aggregated to the residue level for inclusion in the dataset.

First, we collect the set of locations of all atoms in the structure, under all altlocs. Next, we iterate over each residue in the chain, each atom within it, and each altloc defined for that atom. Given the location of this altloc atom as a source, we calculate the distance to each target atom in the set of all locations. Two atoms are defined as in contact when their distance is below a threshold of 5 Angstroms. Each detected contact is then classified into one of three types: regular AA contact, out-of-chain (OOC) contact or ligand contact, depending on the identity and chain of the target atom. Contacts from all atoms of the current residue are collected into one of three lists of contacts for that residue, based on this classification. The minimum distance is calculated across atoms belonging to the same source and target residue. Hydrogen atoms and water molecules are always excluded even if they are modeled in the structure.

### Codon assignment

Since the exact genetic sequence of the protein is not annotated in the PDB we assigned codons from the native sequence following the procedure described in our prior work^17^. Given a PDB chain, we obtain its unique Uniprot ID from the previous step. We query Uniprot to obtain all cross-referenced IDs to the European Nucleotide Archive (ENA). From the ENA database, we obtain all available genetic sequences for the specific protein, translate each genetic sequence to an amino-acid sequence using the standard genetic code table, and perform pairwise sequence alignment between the PDB chain’s amino-acid sequence and the translated genetic sequences. The alignment is performed using BioPython using the same options as in the previous section.

Following the pairwise alignment of the amino acid sequence to all translated genetic sequences, we obtain the aligned codons from each sequence and assign them to corresponding residues from the PDB chain. This process yields zero or more assigned codons per residue in the PDB chain. In cases where there is more than one codon (i.e., different genetic sequences contributed different codons), we choose the most common, and reflect this ambiguity by assigning a codon score which is the proportion of genetic sequences that contributed the assigned codon.

### Removal of low-quality structures

**Fig. 1:**
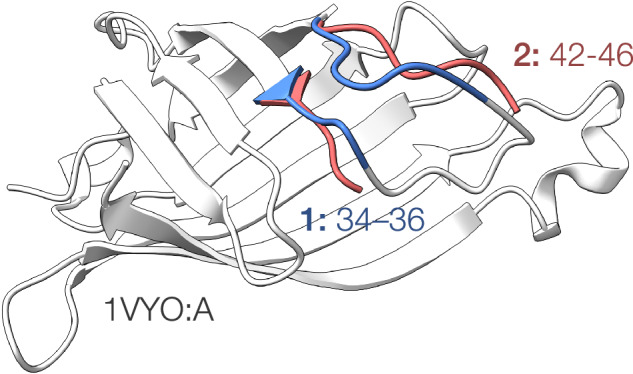
Example of broken altloc chain shown on the crystal structure 1VYO (Avidin at 1.48Å resolution). Altloc B (red) is not modelled between residues 37 and 41 and is therefore counted as two separate segments in our data set.

We used the R-factor to remove structures with a potentially poor fit to the electron density. The intersection of two criteria was used to define a structure as admissible: (1) R_work_ ≤ 0.98 R_free_; and (2) R_free_ ≤ min{0.3, max{0.2, resolution-dependent cut-off}}. The resolution-dependent cut-off was fitted as a monotone polynomial to the 90%-tile of R_free_ estimated in 12 equiprobable resolution bins ranging from 0.5 to 3.5Å (Fig. 1).

### Non-redundant cluster assignment

To account for redundancy of the collected chains and proteins they originate from, we clustered the chains into non-redundant clusters using the amino acid sequence data. Clustering was performed using mmseq2^18^ with minimum sequence identity threshold 0.5 and target coverage 0.8. Cluster identities were recorded in the metadata alongside with the chain identities.

### Segmentation of contiguous altlocs

For each of the collected chains containing altlocs, we grouped all altlocs with contiguous residue numbers into numerically numbered segments. Assignment as a segment requires the altlocs at every location within the segment. This means that in cases of altlocs broken by missing residues, this section will be counted as two segments (Fig. 1)

## Data Records

The dataset is available through Harvard Dataverse^12^. The dataset contains amino acid-level data records and chain-level metadata records.

### Data

A single comma-separated value (csv) file containing local altloc description for the entire dataset can be accessed as altloc_data.csv.

The dataset entries (rows) each represent a single residue in a specific chain of a PDB structure. For each row, columns, describing the residue, are available and are explained in Table 1.

**Table 1:** Description of the column in the data records.

Column Name	Description	Example
pdb_id	PDB and chain identifier, delimited by a colon.	2WUR:A
unp_id	Uniprot protein identifier.	P42212
pdb_idx	Zero-based index of the residue in the PDB chain sequence.	13
unp_idx	Zero-based index of the corresponding residue in the Uniprot sequence.	12
seg_id	Contiguous altloc segment number within the chain.	1
res_name	Residue amino acid name, using the standard single-letter designation.	P
res_icode	Residue insertion code. Sometimes used by PDB authors to represents an experimental addition to the protein sequence. Value of ‘M’ denotes an unmodelled residue.	M
res_hflag	Residue hetero flag. Describes any molecules, besides amino acids and water, which are part of the chain.	H_GYS
rel_loc	Relative location of the residue from the start of the sequence, as a value in the interval (0, 1].	0.24
codon	Codon assigned to this residue by aligning to genetic sequence from ENA.	CCA
codon_score	The proportion of ENA records which contained the assigned codon at the current position. A value of 1.0 means the assignment was unique.	0.8
codon_opts	Names of all codons that were found in ENA records aligned to the current position, delimited by ‘/’.	GGA/GGT
secondary	DSSP-assigned secondary structure label.	E
*phi_X*	The standard $\varphi$ dihedral angle at this residue, calculated by using the altloc X of all atoms involved (where available). Given in degrees within the range (−180, 180].	−123.456
*psi_X*	Same as above, for the standard $\psi$ dihedral angle.	123.456
*omega_X*	Same as above, for the standard $\omega$ dihedral angle.	−179.876
*bfactor_X*	Average b-factor computed from the b-factors of the *X* altloc in the current residue’s backbone atoms (N, CA, C)	6.543
*contact_count_X*	Total number of *X-*contacts (as defined in the preceding sections) between the current residue’s atoms and atoms of other residues in the structure.	42
*contact_types_X*	Type of contacts this residue’s atoms make. Currently not implemented. Always ‘proximal’.	proximal
*contact_smax_X*	Maximum distance, in the amino acid sequence, of any of the current residue’s *X-*contacts.	24
*contact_ooc_X*	Comma-delimited list of *X-*contacts between the current residue’s atoms and atoms of a residue in another chain. Contacts specified as: ‘chain_id:residue_name:residue_index:target_altloc:distance’. Target altloc appears as ~ if target has only one modeled location.	A:T:9:B:3.97, C:M:88:A:4.03
*contact_non_aa_X*	Comma-delimited list of *X-*contacts between the current residue’s atoms and atoms of ligand in the structure. Contact is specified as above, with ligand instead of residue name.	A:GYS:66:~:4.95, A:EOH:242:B:3.98
*contact_aas_X*	Comma-delimited list of *X-*contacts between the current residue’s atoms and atoms of a residue in the same chain. Contacts specified as above.	A:T:9:B:3.97, C:M:88:A:4.03

Where altlocs exist for a particular residue, additional columns are populated within the same row and include information pertaining to each altloc as described in Table 2. Italic characters, *X* and *Y*, in the column names shown in Table 2 are placeholders for actual altloc label names. We limit the altlocs to four (A, B, C, D).

**Table 2:** Description of the columns describing altlocs in the data records.

Column Name	Description	Example
num_altlocs	Number of alternate location labels across all the backbone atoms of the residue.	3
altlocs_N	Semicolon-delimited names of altlocs which exist for the nitrogen atom. May contain optional original altloc label in parenthesis.	A;B(Z)
altlocs_CA	Semicolon-delimited names of altlocs which exist for the alpha-carbon atom. Format as above.	A;B(Z)
altlocs_C	Semicolon-delimited names of altlocs which exist for the carbon atom. Format as above.	A;B(Z)
*dist_CA_XY*	Distance in Angstroms between the alpha-carbon atom positions of this residue, for altloc *X* and altloc *Y*.	0.089
*dist_CA_XY_norm*	Same as above, where the distance is normalized to units of the standard deviation, based on the b-factors of both altlocs.	0.36
*sigma_CA_X*	Standard deviation of atom location, in Angstroms, for altloc *X*. Calculated from the isotropic b-factor as $\sigma =\sqrt{B/8{\pi }^{2}}$.	0.235
n_terminal_dist	Distance in number of residues from the N-terminal. Note: if terminal regions contain unmodelled residues, the distance is under-estimated.	10
c_terminal_dist	Distance in number of residues from the C-terminal. Note: if terminal regions contain unmodelled residues, the distance is under-estimated.	10

### Metadata

A single comma-separated value (csv) file containing PDB structure- and chain-level metadata can be accessed as altloc_metadata.csv.

The dataset entries (rows) each represent a single chain. Columns are described in Table 3.

**Table 3:** Description of the column in the metadata records.

Column Name	Description
pdb_id	PDB and chain identifier, delimited by a colon.
unp_id	Uniprot protein identifier.
ena_id	Identifier of ENA genetic sequence used for codon assignment.
seq_len	Number of residues in PDB chain
num_altlocs	Number of residues which harbor altlocs in the PDB chain.
title	Title (as deposited in the PDB) of the structure containing this chain.
description	Description (as deposited in the PDB) of the structure containing this chain.
entity_description	Description (as deposited in the PDB) of the polymer entity associated with this chain.
deposition_date	Date the structure was deposited to the PDB.
entity_source_org	Name of the protein’s source organism.
entity_source_org_id	NCBI taxonomy ID of the source organism.
entity_host_org	Name of the host organism, i.e. the organism in which the protein was experimentally expressed.
entity_host_org_id	NCBI taxonomy ID of the host organism.
resolution	X-ray crystallography high resolution limit of data collection.
resolution_low	X-ray crystallography low resolution limit of data collection.
r_free	Structure refinement R_free_ value.
r_work	Structure refinement R_work_ value.
space_group	Symbol of space-group describing the crystal symmetries.
cg_ph	The pH at which the crystal was grown.
cg_temp	The temperature in kelvins at which the crystal was grown.
chain_ligands	List of the chemical component identifiers for all ligand interactions in the chain.
ligands	List of the chemical component identifiers for all ligand interactions in the structure.
entity_chains	PDB chain identifiers of all chains in the structure which belong to the same polymer entity as the current chain.
entity_auth_chains	As above but using the PDB deposition author’s original chain identifiers instead of the PDB-assigned identifiers.
chain_entities	Identifier (internal to the structure) of the polymer entity associated with this chain.
chain_to_auth_chain	Deposition author-assigned name of this chain.
entity_sequence	Canonical sequence of the protein in the standard one-letter code of amino acids.
num_altloc_segments	The number of segments of contiguous residues harboring altlocs within the chain.
cluster_id	The mmseq2 cluster identity of the current chain sequence.

## Technical Validation

**Fig. 2:**
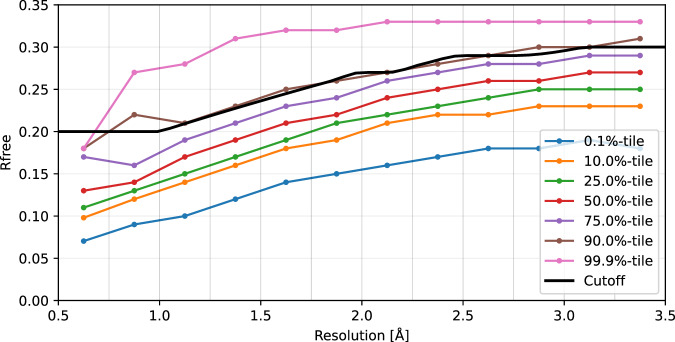
Quantiles of the R_free_ parameter across the collected structures as a function of resolution. The cut-off above which low-quality structures were rejected is plotted in thick black.

Since our data is derived from the PDB^13^, our starting point are records that have already been validated through the standardised procedures of this well-established global repository. The quality and reliability of crystallographic structures is commonly assessed by the R-factor that measure of the goodness of fit between the model and the experimental X-ray diffraction data for the refined data. A small percent of data is left out of refinement and an analogous R_free_ is calculated to assess against the R factor for potential overfitting biases. Figure 2 shows the resolution-dependent R_free_ thresholds we used to exclude models with a poor fit the experimental data.

**Fig. 3:**
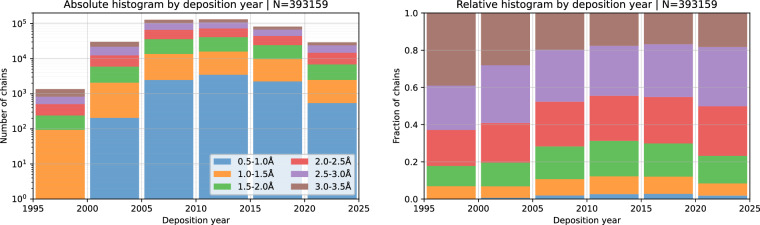
Absolute and relative histograms of the resolution of the retained structures by structure deposition date.

**Fig. 4:**
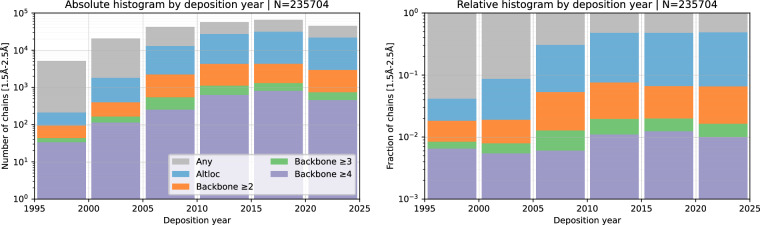
Absolute and relative histograms of backbone altloc segment lengths in the resolution range 1.5–2.5Å by structure deposition date. *Any* refers to any of the collected chains regardless of altloc presence; *Altloc* refers to the presence of any altloc; Backbone ≥ n refers to the presence of a segment of length ≥ n containing alternate locations for CA atoms. We observe a steady and sharp increase in the fraction of structures with modelled altlocs in years preceding 2010 which we attribute to the improvement of experimental techniques and data analysis software. Note that the fraction of structures with long altloc segments (≥3) increases only slightly, probably since such segments are more readily modelled with older protocols.

**Fig. 5:**
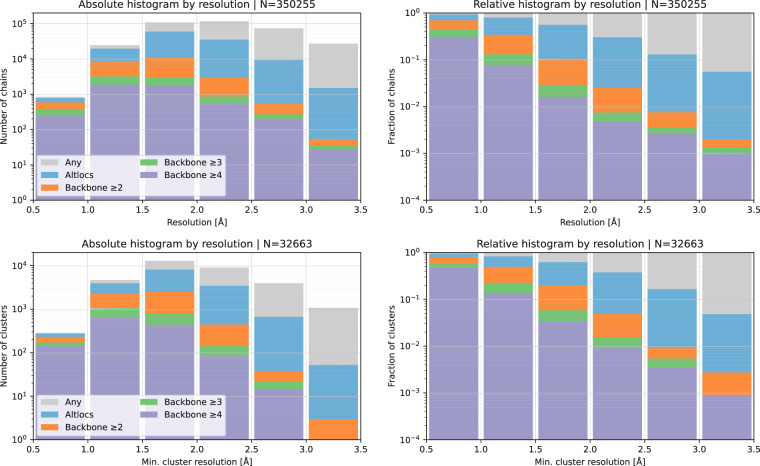
Absolute and relative histograms of backbone altloc segment lengths by resolution. *Any* refers to any of the collected chains regardless of altloc presence; *Altloc* refers to the presence of any altloc; Backbone ≥ n refers to the presence of a segment of length ≥ n containing alternate locations for CA atoms. Shown are distributions of individual chains (first row), and non-redundant clusters (second row). Note that the fraction of modelled altloc segments (especially the long ones) consistently increases with better resolution. We attribute this trend to the finer ability to discern between truly multi-modal distribution of the electron density (altlocs), and the uni-modal high B-factor case. About 4% of segments of length 2 are peptide flips.

As a validation step, we verified that our collected data follows expected trends with relation to altloc abundance. Figure 3 shows the distribution of resolutions for structures deposited in PDB during different time brackets showing that structures of resolutions between 1.5A and 2.5A constituted a large and similar fraction of structures deposited during the period since 1995. Focusing on this resolution range, Fig. 4 shows how the fraction of structures modelled with an altloc, particularly short segments of 1–2 amino acids, progressively increased until 2010. This rise would be expected as modelling refinement techniques improved and became more automated^19,20^. Another clear and expected trend is the increase in altlocs with resolution as shown in Fig. 5.

**Fig. 6:**
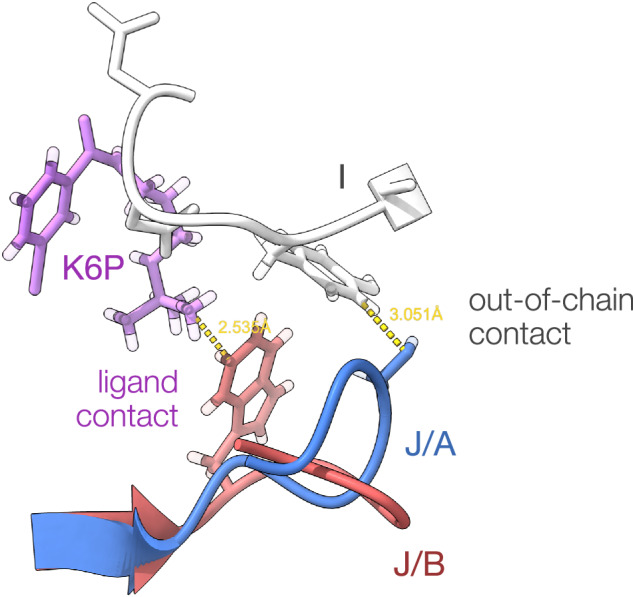
Categorization of contact types shown on the crystal structure 6MXX (TP53-binding protein 1 at 2.3Å resolution). Two well-separated 7 amino acid-long altloc segments in chain J (residues 1493–1499, displayed in red and blue) are in proximity of Y1523 in chain I (out-of-chain contact displayed in white), and K6P ligand molecule (ligand contact displayed in violet). The records corresponding to the highlighted contacts are contact_ooc_A[J:S:1497] =  "I:Y:1523:~:3.05,I:E:1524:~:4.32" contact_non_aa_B[J:W:1495] = "J:K6P:1701:A:2.54,J:K6P:1701:B:4.13" Note that hydrogens atoms (displayed with transparency) are excluded from distance calculations.

Manual verification of the correspondence between collected contact distances and those observed in visualisation of the protein structure was carried out as shown in Fig. 6. We note here that our current dataset analyses the contents of the asymmetric unit cell as these coordinates are explicitly available with the PDB file. A current limitation of this dataset is that it does not identify contacts made within the crystal lattice.

## Data Availability

The code implementing the described data collection and analysis methods is accessible at https://github.com/vistalab-technion/pp5.
